# The influence of basic ventilation strategies and anesthetic techniques on cerebral oxygenation in the beach chair position: study protocol

**DOI:** 10.1186/1471-2253-12-23

**Published:** 2012-09-20

**Authors:** Paul Picton, Andrew Dering, Bruce Miller, Amy Shanks, George A Mashour

**Affiliations:** 1 Department of Anesthesiology, University of Michigan Medical School, Ann Arbor, Michigan, USA; 2 Department of Orthopedic Surgery, University of Michigan Medical School, Ann Arbor, Michigan, USA

## Abstract

**Background:**

Beach chair positioning during general anesthesia is associated with a high incidence of cerebral desaturation; poor neurological outcome is a growing concern. There are no published data pertaining to changes in cerebral oxygenation seen with increases in the inspired oxygen fraction or end-tidal carbon dioxide in patients anesthetized in the beach chair position. Furthermore, the effect anesthetic agents have has not been thoroughly investigated in this context. We plan to test the hypothesis that changes in inspired oxygen fraction or end-tidal carbon dioxide correlate to a significant change in regional cerebral oxygenation in anesthetized patients in beach chair position. We will also compare the effects that inhaled and intravenous anesthetics have on this process.

**Methods/design:**

This is a prospective within-group study of patients undergoing shoulder arthroscopy in the beach chair position which incorporates a randomized comparison between two anesthetics, approved by the Institutional Review Board of the University of Michigan, Ann Arbor. The primary outcome measure is the change in regional cerebral oxygenation due to sequential changes in oxygenation and ventilation. A sample size of 48 will have greater than 80% power to detect an absolute 4-5% difference in regional cerebral oxygenation caused by changes in ventilation strategy. The secondary outcome is the effect of anesthetic choice on cerebral desaturation in the beach chair position or response to changes in ventilation strategy. Fifty-four patients will be recruited, allowing for drop out, targeting 24 patients in each group randomized to an anesthetic. Regional cerebral oxygenation will be measured using the INVOS 5100C monitor (Covidien, Boulder, CO). Following induction of anesthesia, intubation and positioning, inspired oxygen fraction and minute ventilation will be sequentially adjusted. At each set point, regional cerebral oxygenation will be recorded and venous blood gas analysis performed. The overall statistical analysis will use a repeated measures analysis of variance with Tukey’s HSD procedure for post hoc contrasts.

**Discussion:**

If simple maneuvers of ventilation or anesthetic technique can prevent cerebral hypoxia, patient outcome may be improved. This is the first study to investigate the effects of ventilation strategies on cerebral oxygenation in patients anesthetized in beach chair position.

**Trial registration:**

NCT01535274

## Background

Poor neurological outcome in otherwise healthy patients following anesthesia in the beach chair position is a significant concern [1] and has been reported in patients with recorded blood pressures that many anesthesiologists would consider acceptable [2].

There are compelling arguments for the application of a non-invasive monitor of cerebral blood flow, function and/or oxygenation for patients placed in the beach chair position. The measurement of regional cerebral oxygenation (rSO_2_) has been widely used for patients undergoing cardiac [3] and vascular surgery [4,5] and, more recently, in anesthetized patients undergoing surgery in beach chair position [6] where it has revealed a high incidence of severe cerebral desaturation events [7]. Even patients undergoing arthroscopic shoulder surgery in the beach chair position with regional anesthesia (brachial plexus block) and sedation alone have a 10% chance of experiencing severe cerebral desaturation [8].

The use of cerebral near-infrared spectroscopy (NIRS) allows for the continuous non-invasive monitoring of rSO_2_ by measuring the relative concentrations of oxyhemoglobin and deoxyhemoglobin within the field of view and, therefore, provides an estimate of the balance between cerebral oxygen supply and demand [9]. Recent reports in conscious volunteers [10] and anesthetized patients without vascular disease [11] demonstrate a relationship between inspired oxygen fraction (FIO_2_) and end-tidal carbon dioxide (PETCO_2_) with cerebral oxygenation.

Observational data suggest a relationship between PETCO_2_ and rSO_2_ specifically in patients anesthetized in the beach chair position [12] and mechanical ventilation may have negative influence on cerebral auto-regulation [13]; as one of the primary determinants of set minute ventilation, PETCO_2_ must be considered. Furthermore, different anesthetic agents have distinct effects on cerebral hemodynamics and metabolism [14,15], yet there has been no direct comparison of the effects of anesthetic agents on rSO_2_ in the beach chair position. The effect on cerebral oxygenation resultant upon any interaction between inspired gas composition and anesthetic choice will be of interest.

The study described here will test the hypothesis that the manipulation of inspired oxygen fraction and end tidal carbon dioxide leads to significant changes in rSO_2_ in patients anesthetized in the beach chair position. The influence of anesthetic technique on this process will be tested as a secondary outcome.

## Methods/design

**Figure 1:**
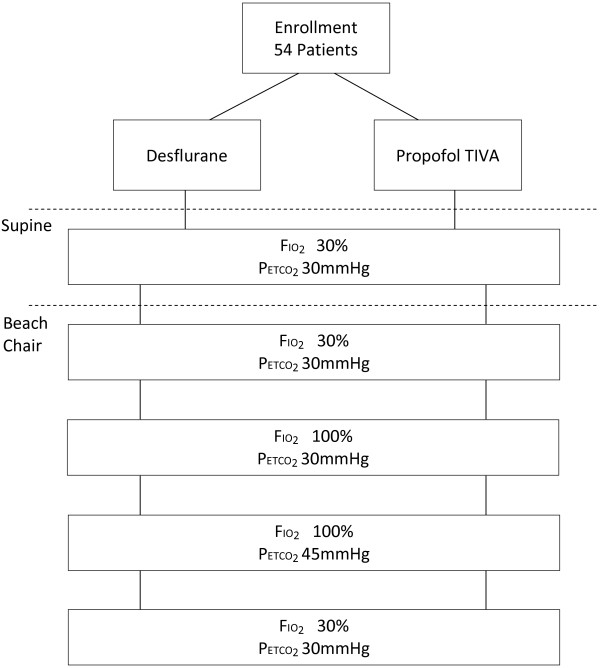
Figure illustrating the overall study design.

This is a prospective within-group study with an additional randomized comparison of two anesthetic regimens (Figure 1). The study was approved by the Institutional Review Board of the University of Michigan, Ann Arbor. The primary aim is to determine the effect of increasing the FIO_2_ or PETCO_2_ on cerebral oxygen saturation in patients anesthetized in the beach chair position. Our secondary aim is to investigate the effects of differing anesthetic agents on cerebral oxygenation in patients placed in the beach chair position.

Patients who are scheduled for elective arthroscopic shoulder surgery in the beach chair position under general anesthesia with supplemental interscalene block will be recruited following written informed consent. All surgeries will be conducted by a single surgeon and single anesthesiologist at a “stand-alone” ambulatory surgery facility. Patients will be excluded if they refuse to give consent, are ineligible for interscalene block, have a history of cardiovascular disease, cerebrovascular disease, hypertension (either by diagnosis, pharmacologic treatment as a surrogate, or a blood pressure measured in the surgical preoperative clinic of greater than 140/90), suffer from respiratory failure, or do not speak English. Recruited patients will undergo computer-generated randomization to receive desflurane or total intravenous anesthesia (TIVA) with propofol for maintenance of anesthesia. These agents were chosen because they have differential effects on cerebral vasodilation, auto-regulation and CO_2_ responsiveness [15-17]. Patients and data analysts will be blinded to the anesthetic choice; the anesthesiologist caring for the patient will be blinded to cerebral oximetry values. All patients will have an interscalene block placed before induction of anesthesia by a single anesthesiologist. Patients will be pre-medicated with midazolam 0.5-2 mg. Following pre-oxygenation, anesthesia will be induced using fentanyl (1–2 mcg/kg) and propofol (0.5-2 mg/kg). Muscle relaxation will be initiated and maintained with an appropriate combination of succinylcholine and non-depolarizing muscle relaxants as deemed necessary by the anesthesiologist. The patient’s trachea will be intubated and their lungs ventilated. General anesthesia will be maintained as dictated by randomization. Depth of anesthesia will be assessed by Bispectral Index (BIS; Covidien, Boulder, CO) and be controlled within the range 40–60 for all agents. Nitrous oxide could possibly confound results and will therefore not be used.

There may be an advantage of ephedrine over phenylephrine in preserving rSO_2_ when these drugs are used as a bolus for the management of hypotension [18]. Phenylephrine given by bolus is associated with a 2.8% reduction in rSO_2_ of approximately 8 min duration whereas ephedrine is not associated with any such change [19]. The relative effect of these agents when used as infusions has not been published and phenylephrine has been used as an infusion in other studies examining rSO_2_. Here, we will use ephedrine (5 mg) in preference to phenylephrine (50-100mcg) as an intravenous bolus medication for the treatment of intraoperative hypotension when heart rate is less than 80 beats per minute. Phenylephrine by infusion (200mcg/ml) will be titrated to maintain blood pressure within 20% of preoperative mean arterial pressure (MAP) when there has been a requirement for more than 400mcg of phenylephrine over a 20 min period. Pre-treatment with ephedrine significantly blunts the decrease in rSO_2_ seen with bolus phenylephrine for at least 20 min [19]. If phenylephrine bolus dose is used as the first treatment for intraoperative hypotension we will delay the recording of results following a trial intervention by at least 8 min.

For MAPs greater than 70-80 mmHg associated with intraoperative bleeding the initial treatment will be to ensure adequate depth of anesthesia (BIS 40–60), followed by administration of incremental boluses of fentanyl 25mcg, up to a maximum dose of 2.5 mcg/kg. Failure of this to achieve the required reduction in blood pressure will result in the administration of the antihypertensive medications labetolol or hydralazine at the discretion of the patient’s anesthesiologist.

Standard American Society of Anesthesiologists monitoring will be used for all patients. The BIS Quatro electrode will be placed diagonally on the patient’s left forehead. Cerebral oxygenation will be measured using the INVOS 5100C monitor (Covidien, Boulder, CO). Optodes will be applied, before induction of anesthesia, by a single trained researcher on either side of the BIS Quatro sensor as recommended by the manufacturer.

Following induction of anesthesia, FIO_2_ and minute ventilation will be sequentially adjusted to achieve:

1) FIO_2_ 30% (70% nitrogen), PETCO_2_ 30 mmHg – supine position.

2) FIO_2_ 30% (70% nitrogen), PETCO_2_30mmHg – beach chair position.

3) FIO_2_ 100%, PETCO_2_ 30 mmHg – beach chair position.

4) FIO_2_ 100%, PETCO_2_ 45 mmHg – beach chair position.

5) FIO_2_ 30%, PETCO_2_ 30 mmHg – beach chair position.

Minute ventilation will be adjusted by changing respiratory rate rather than manipulating tidal volume. Starting tidal volume will be set at 6–8 cm^3^/kg. The first measurement point in beach chair position will be obtained 15 min after positioning (at which point the maximal decrease in cerebral oxygen saturation is observed to occur [7]) or immediately if severe cerebral desaturation (absolute value rSO_2_ < 55% or a decrease from baseline of ≥ 20%) is sustained for ≥ 3 min in either hemisphere. It has been shown that the change in rSO_2_ is complete and stable within 5 min following a change in inspired gas composition [4]. Thus, after 5 min at each subsequent set point, rSO_2_ will be recorded and venous blood gas analysis performed. If a patient’s pulse oximetry reading is persistently <95% at FIO_2_ 30% a higher FIO_2_ will be used at the discretion of the patient’s anesthesiologist, the patient will be withdrawn from the study and an additional patient will be recruited. All sustained cerebral desaturation events, as defined above, will be recorded and communicated to the anesthesiologist to allow intervention as deemed appropriate by the anesthesiologist. Data will be recorded as a “snap-shot” at the time of venous blood gas analysis. A separate intravenous cannula will be placed into a peripheral vein for this purpose. Demographic, intra-operative and outcome data will be retrieved from the patient’s electronic anesthetic and medical records.

### Statistical considerations

#### Power

The reported mean rSO_2_ is 67.1% ± 6.2 for patients placed in beach chair position [7]. Based on a previous investigation [11], we expect a 6–8 percentage point difference in rSO_2_ resultant upon the planned change in FIO_2_ and a 2–4 percentage point difference resultant upon the planned change in PETCO_2_. A total percentage point increase in excess of 10 would be of clinical significance. A sample size of 48 will have a power of greater than 0.8 to detect a 4-5% in the planned pairwise comparisons. Allowing for an approximately 10% drop out 54 subjects will be recruited, targeting 24 patients in each group randomized to an anesthetic. The power for the comparison between the two anesthetic regimens is better than 85% for a difference of 6% which would be a clinically important difference for the secondary outcome.

#### Analysis

Data will be analyzed with a repeated measures two-way analysis of variance in which ventilation strategy is the within-subjects factor and anesthetic regimen is the between-subjects factor. Residuals will be assessed for normality and equal variances; if necessary, the analyses will be adjusted. The primary analysis will focus on the comparison between the ventilation strategies. A post hoc Tukey’s HSD procedure will be used to correct for all pairwise comparisons between ventilation strategies.

The effect of desflurane or propofol on cerebral desaturation and a possible differential response to changes in ventilation strategy will be evaluated as a secondary outcome measure. Analysis for the primary outcome will be adjusted if interaction effects between ventilation strategy and the anesthetic used are significant. A p value of <0.05 will be considered statistically significant.

## Discussion

Ensuring the safety of patients anesthetized in the beach chair position is of widespread and significant interest. The simple modulation of inspired gas composition has proven to reliably improve cerebral oxygenation measured by NIRS in normal awake subjects [10], healthy supine anesthetized patients [11] and patients undergoing carotid endarterectomy with either regional or general anesthesia [4,5]. The major aim of the proposed study is to determine the best ventilation strategy to maintain cerebral oxygenation and limit neurological risk in patients anesthetized in the beach chair position. We will investigate the response, the magnitude of the response and the direction of the response in rSO_2_ resultant upon changes in FIO_2_ and PETCO_2_.

Controversy exists regarding the effect of beach chair position and anesthesia on cerebral oxygenation and its measurement. Although it has been suggested that cerebral oxygenation should be monitored in all patients anesthetized in the beach chair position [20] this is certainly not yet a standard of care. While severe cerebral desaturation has been effectively measured using the INVOS series of cerebral oximeters [12] similar findings are not evident when NIRO instruments are used [21] despite the fact that both technologies are based on NIRS. Invasive jugular venous bulb saturation (Sjv0_2_) may be a more sensitive technique for the detection of cerebral desaturation [22], but unless routine shoulder surgery is relocated from specific ambulatory surgery centers where invasive techniques are neither available nor encouraged, one must first consider a non-invasive solution.

In terms of anesthetic choice, the balance between cerebral blood supply and oxygen demand is our main concern. Propofol proportionally decreases cerebral blood flow (CBF) and cerebral metabolic rate for oxygen (CMRO_2_) without affecting cerebral ateriovenous oxygen difference [23]. Desflurane causes a reduction in CMRO_2_ but the expected reduction in cerebral blood flow is overcome by direct cerebral vasodilation. The decrease in CMRO_2_ and cerebral vasodilation are greater with desflurane when compared to other inhalational agents [15]. Regional oximetry values increase in supine patients with increasing anesthetic depth of desflurane [24] and cerebral desaturation is more frequently observed during one lung ventilation while using TIVA with propofol compared to inhalational anesthesia [14]. Cerebral oxygenation is reported to be better preserved with sevoflurane-nitrous oxide anesthesia when compared to propofol-remifentanil anesthesia for patients placed in beach chair position [22], but nitrous oxide and remifentanil may have acted as uncontrolled confounding factors. Here we will compare desflurane to TIVA with propofol and control all other aspects of anesthesia. In conclusion, the proposed study is the first to investigate specifically the effects of ventilation strategies and specific anesthetic agents on cerebral oxygenation in patients anesthetized in the beach chair position.

## Competing interests

The authors declare that they have no competing interests.

## Authors’ contributions

PP and GM conceived of the study. All authors have contributed to study design and manuscript preparation. All authors have read and approved the final manuscript.

## Funding

The study will be funded by the Department of Anesthesiology at the University of Michigan. The INVOS 5100C cerebral oxygenation and BIS monitor will be loaned and cerebral oxygenation optodes will be provided at no cost by the manufacturer (Covidien, Boulder, CO). The manufacturers have had no role in the study design and no role in the preparation of this manuscript.

## Pre-publication history

The pre-publication history for this paper can be accessed here:

http://www.biomedcentral.com/1471-2253/12/23/prepub
